# Urgent Vitrectomy with Vancomycin Infusion, Silicone Oil Endotamponade, and General Antibiotic Treatment in Multiple Cases of Endophthalmitis from a Single Day of Intravitreal Injections—Case Series

**DOI:** 10.3390/jcm10051059

**Published:** 2021-03-04

**Authors:** Agata Pietras-Baczewska, Ewa Jasińska, Mario Damiano Toro, Vincenza Bonfiglio, Michele Reibaldi, Teresio Avitabile, Katarzyna Nowomiejska, Robert Rejdak

**Affiliations:** 1 Department of General Ophthalmology, Medical University of Lublin, 20-059 Lublin, Poland; Ewjasisnka@gmail.com (E.J.); toro.mario@email.it (M.D.T.); katarzyna.nowomiejska@umlub.pl (K.N.); robert.rejdak@umlub.pl (R.R.); 2 Faculty of Medicine, Collegium Medicum Cardinal Stefan Wyszyński University, 01-815 Warsaw, Poland; 3 Department of Experimental Biomedicine and Clinical Neuroscience, Ophthalmology Section, University of Palermo, 90133 Palermo, Italy; enzabonfiglio@gmail.com; 4 Department of Surgical Sciences, Eye Clinic Section, University of Turin, 10124 Turin, Italy; mreibaldi@libero.it; 5 Department of Ophthalmology, University of Catania, 95124 Catania, Italy; t.avitabile@unict.it

**Keywords:** post-injection endophthalmitis, bacterial endophthalmitis, *Streptococcus mitis/oralis*, endophthalmitis treatment

## Abstract

The aim of this study was to report on the anatomical and functional results of surgical management of seven cases of endophthalmitis related to a single day of intravitreal aflibercept injections. Patients with signs of endophthalmitis who underwent aflibercept injections (seven eyes) performed on the same day were retrospectively evaluated. The data of visual acuity and optical coherence tomography (OCT) within nine months of the follow-up and the treatment and results of microbiological cultures are reported. Four of the total seven cases had a positive bacterial culture outcome (*Streptococcus mitis)*. All patients underwent vitrectomy combined with phacoemulsification when the eyes were not pseudophakic, vancomycin infusion, and silicone oil tamponade within 24 h; additionally, systemic antibiotics were administered intravenously. The final best-corrected visual acuity (BCVA) after the treatment was finger counting or light perception in all cases, and all eyes were saved with disruption of the inner retinal layers and stabilization of the retina in regard to changes related to the wet age-related macular degeneration (AMD). Although the retinal anatomy was mostly preserved, most of the patients affected by *Streptococcus mitis*-induced endophthalmitis did not regain baseline vision after the therapy.

## 1. Introduction

Endophthalmitis is a serious and devastating condition, which may lead to irreversible blindness in the affected eye. It is a purulent inflammation of the intraocular fluids, i.e., the vitreous and the aqueous humor. Although the etiology of this condition can be both endogenous and exogenous, it is mostly secondary to intraocular surgeries, injections, or penetrating ocular trauma [1]. Acute endophthalmitis appears as a postoperative complication within 1–2 weeks after the surgical intervention, most commonly on the third to fifth postoperative day [2]. The literature describes that endophthalmitis affects approximately 0.02–0.03% of patients after intraocular injection [1].

Since 2004, the number of intraocular injections performed yearly has increased and they have now become the most common procedure in ophthalmological treatment [3]. The number of indications for anti-vascular endothelial growth factor (anti-VEGF) injections (ranibizumab, bevacizumab, and aflibercept) is growing worldwide. Despite ample evidence of the safety of intravitreal injections, each subsequent injection carries the risk of complications. Although the risk is low, the visual consequences can be devastating [4].

*Staphyloccocus epidermidis* is the most common pathogen causing endophthalmitis as a complication after cataract surgery [1]. It is part of the physiological bacterial flora of human skin, which can be transferred into the eye during ophthalmologic procedures. *S. epidermidis* is now described as responsible for a majority of intra-hospital infections. *Streptococcus mitis/oralis*, which causes endocarditis, meningitis, and endophthalmitis, as shown in the literature, are less commonly present in the physiological flora [5,6].

They are facultatively anaerobic catalase-negative, Gram-positive cocci. They represent the oral streptococcal group (mitis phylogenetic group) and are regarded as opportunistic pathogens of the human oral cavity, oropharynx, and gastrointestinal tract and are a frequent etiological factor of infections in immunocompromised patients [7]. These species are the second most common bacterial isolates identified in cases of endophthalmitis after intravitreal injections [8]. Their strains share over 99% of 16S rRNA sequence identity with *S. pneumoniae*; however, the DNA similarity values for the entire chromosome are estimated at less than 60%. *S. mitis* and *S. oralis* have competence for natural genetic transformation [9].

Vancomycin is a glycopeptide antibiotic applied in the treatment of infections caused by Gram-positive bacteria that are unresponsive to other antibiotics [10]. This antibiotic is used in the treatment of endophthalmitis as an intravitreal injection alone or at the end of pars plana vitrectomy [11].

The aim of this article was to describe a series of seven patients who developed acute endophthalmitis after intraocular injections performed on the same day, further surgical and pharmacological management, and the treatment outcomes.

## 2. Materials and Methods

This retrospective observational study describes seven consecutive patients with endophthalmitis after aflibercept injection treated at the Department of General Ophthalmology of the Medical University of Lublin, Poland, in January 2020. The study followed the tenets of the Declaration of Helsinki. Approval of the Ethics Committee at the Medical University of Lublin was obtained. The patients gave their written informed consent.

The inclusion criteria were clinical manifestations of acute endophthalmitis within 48 h after intraocular aflibercept injection performed on the same day. The injections were all performed by one surgeon on the same day in the regional hospital. All patients received antibiotic prescriptions after the injection. The patients claimed they all used the drops as prescribed and followed the doctor’s instructions and orders. However, we had no ability to verify the patients’ statements. The data included the age and gender of the patients, the pre- and postoperative best-corrected visual acuity (BCVA; Snellen charts), intraocular pressure (IOP), microbiological culture, treatment method, and status of the final anterior segment and retina.

When the patients’ BCVA was less than 0.1 (in Snellen charts), hand movement (HM), finger counting (FC), light perception (LP), or no light perception (NLP) were used to describe the visual acuities. Additionally, slit-lamp examination of the anterior segment and fundus examination with fundus photography and optical coherence tomography were performed. Follow-up examinations were performed on the first postoperative day, after two weeks, and after two, six, and nine months.

### 2.1. Surgical Procedure of Injection

All intraocular injections were performed with local anesthesia with proparacaine solution in the sterile conditions of the operating room. The surgeon and the nursing staff were wearing facial surgical masks and hats. Prior to the intervention, Betadine was administered onto the periocular skin and into the conjunctival sac for one minute for asepsis. At the end of the procedure, the eye was rinsed with Betadine again and levofloxacin drops were administered. One bottle of each type of drop (anesthetic, Betadine, and levofloxacin) was used for all of the patients. A five-day topical antibiotic application was recommended to the patients.

**Figure 1:**
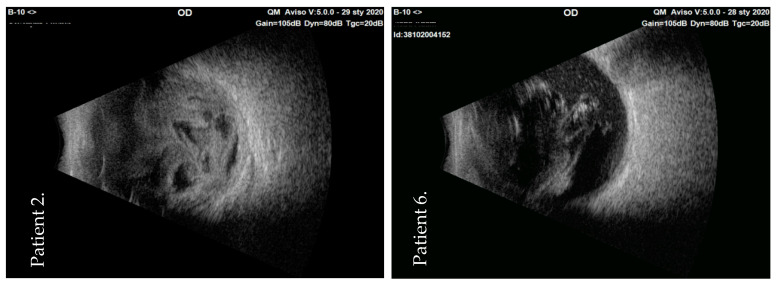
B-scan of ultrasonography images—acute inflammation of the vitreous in the initial examination of two patients (numbers 2 and 6) with post-injection endophthalmitis. OD: oculi dextri.

After the clinical examination (including B-scan ultrasound) (Figure 1) and diagnosis of endophthalmitis, the patients were referred to the Department of General Ophthalmology in Lublin for further treatment.

### 2.2. Surgical Treatment of Endophthalmitis

The surgeries were performed in local peri-bulbar anesthesia. The vitrectomies were all performed by the same experienced surgeon.

The Constellation System (Alcon Laboratories, Inc., Fort-Worth, TX, USA) was used in all cases for 23 G vitrectomy. Before the vitrectomy itself, a vitreous sample was acquired by aspiration for microbiological examination. Cataract surgery with intraocular lens (IOL) implantation was performed in six of the seven eyes before vitrectomy, as these eyes were phakic. Following the specimen collection and central vitreous removal, the core vitrectomy was performed in each surgery.

Ringers’ solution with vancomycin was used in the infusion line throughout the whole vitrectomy procedure. The vancomycin solution was prepared in the operating theatre by dissolving 1 g of Vancomycin in 10 mL of sterile normal (0.9%) saline and then dissolving 1 mL of this mixture in a sterile bottle of 500 mL of infusion fluid [12]. A complete vitrectomy with peripheral shaving and indentation was performed. Subsequently, pathological membranes and bacterial conglomerates were removed together with the peripheral vitreous. Once the retina was clearly visible, endolaser treatment was performed around the retinal breaks if there were any present. The last element before completing the surgery was the intraocular tamponade. The sclerotomies typically did not require sutures for closing.

All patients received a separate set of pharmacological treatments during the postoperative period. Topical antibiotics, i.e., levofloxacin and dexamethasone drops, were administered every hour and 1% atropine solution was applied twice a day. Additionally, all patients had oral antibiotic treatment with levofloxacin for seven days. Dexpanthenol gel was used in cases of corneal epithelium abrasion. Postoperative medication and patient home recommendations included topical steroids and levofloxacin six times a day for one month.

### 2.3. Microbiological Examination

The microbiological diagnostics were performed in accordance with the procedure in force in the hospital laboratory. The vitreous body was collected during the vitrectomy onto a transport medium and immediately sent to the laboratory. The samples were incubated in Biomerieux’s BactAlert (Organon Teknika Corporation, Durham, NC, USA) for five days. When the samples showed signs of microbial growth, they were cultivated on microbial media, i.e., blood agar, MacConcey, Chapman, and Sabouraud, to identify the pathogens. The antibiograms of the cultured bacteria were determined using a Vitek Biomerieux device (Inc., Hazelwood, MO, USA).

## 3. Results

The first signs of endophthalmitis appeared approximately 24 h after the intraocular injection. The patients underwent the vitrectomy approximately 48 h after the first intraocular intervention (range: 36–72 h). The patient group comprised four males and three females, and their average age was 71 years old (range: 56–85 years old) (Table 1). There was a suspicion that the nurses in the operation theatre did not wear face masks during preparation for the surgery. The ultrasound examination was performed in all patients to visualize the inflammatory process in the vitreous cavity. Figure 1 shows the initial ultrasound images of two patients (patient 2 and patient 6).

All patients were managed with pars plana vitrectomy by the same experienced surgeon. Six of the seven patients underwent cataract surgery (phacoemulsification) together with the emergency vitrectomy, and only one patient in the group was pseudophakic. Corneal epithelium abrasion was performed in four cases due to bad view. Anterior chamber rinsing was performed together with vitrectomy in all cases. In six of the seven cases, silicone oil was used as a permanent endotamponade.

In one case, at first, there was a decision to endotamponade the eye with a balanced salt solution (BSS). The decision to endotamponade the eye with the BSS fluid was made according to the current eye status. This was the first patient from the series admitted to our department with moderate intraocular inflammation and a favorable prognosis. However, in the short postoperative period, there was a need for the second surgery with silicone oil endotamponade due to the resumption of the inflammatory process. 

The microbiological examination was positive in four of the seven cases, and *Streptococcus mitis/oralis* were cultured. There was no bacterial growth in the other samples.

All of the patients received their injections from one drug series. The drug lot and remaining syringes were cultured and had a negative result in all of the samples. The operating room equipment was also examined for contamination, and a positive culture was obtained from the patient’s bed and the surgeon’s seat.

BCVA on the admission day included no light perception in one case, light perception in four cases and hand movement in two cases. After the treatment, at the last control examination of BCVA, three patients had hand movement, three counted fingers in front of the eye, and one counted fingers from a 0.5-m distance. The average IOP was 27 mmHg (range 16–44 mmHg) on the admission day, 5 mmHg (range 3–9 mmHg) on the discharge day, and 14 mmHg (range 10–7 mmHg) at the final control examination.

**Figure 2:**
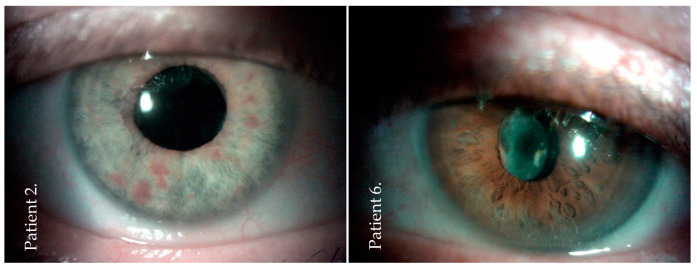
Slit-lamp image of the anterior segment at the final examination (after six months) of two patients (patients: numbers 2 and 6) after post-injection endophthalmitis. No conjunctival irritation but a clear cornea and a clear anterior chamber were observed. There was posterior capsule opacification in patient number 6.

The anterior segment slit-lamp examination after six months showed no complications in four cases and different complications in the other three patients (Table 1). One patient had keratic precipitates, one developed posterior synechiae, and the last one had posterior capsule opacification (Figure 2).

**Figure 3:**
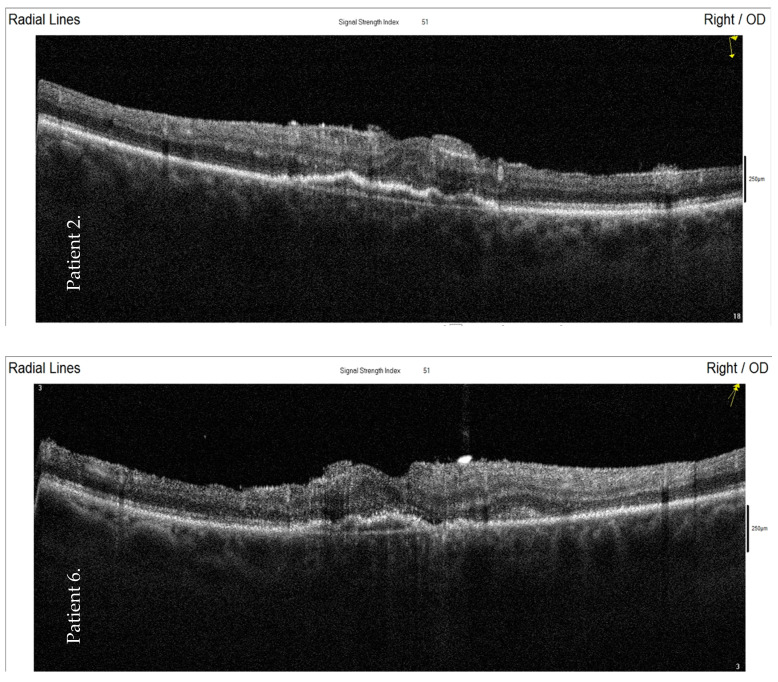
Optical coherence tomography (OCT) images—the final examination of two patients (numbers 2 and 6) four months after the post-injection endophthalmitis and emergency pars plana vitrectomy. The OCT scans show a disruption of the inner retinal layers, an irregular line of the retinal pigment epithelium, and no intraretinal fluid. OD: oculi dextri.

The final fundus examination showed that the retina remained attached in all cases (Table 1). The optical coherence tomography (OCT) scans showed disruption of the inner retinal layers, an irregular line of retinal pigment epithelium, and no intraretinal fluid (Figure 3).

## 4. Discussion

The article describes a series of cases of endophthalmitis after intravitreal injections. *Streptococcus mitis/oralis* were identified as the etiological factor in four of the seven cases. All the patients were treated with immediate vitrectomy with vancomycin infusion and silicone oil tamponade. The optical coherence tomography (OCT) scans showed a preserved retinal anatomy postoperatively; however, the functional results were poor.

*Streptococcus* species have been reported as the causative agents of endophthalmitis after intravitreal injections. The complication is diagnosed more frequently than endophthalmitis after other intraocular surgeries, as reported in the literature [13]. In her review, Durand described that the microbiological cultures of post-injection endophthalmitis included coagulase-negative *Staphylococci* (65%), viridans *Streptococc*i (30%), *S. aureus* (0% to 5%), and others (0% to 4%). In comparison with other ophthalmic surgeries, the author linked the higher endophthalmitis incidence caused by oral flora viridans *Streptococci* with the site of administration of the injections.

As shown recently, the use of laminar airflow can supersede the need for sterile conditions in the operating room during the administration of intravitreal injections [1]. There is no evidence supporting the beneficial role of topical antibiotic prophylaxis after intraocular injection [14]. However, extensive topical antibiotic prophylaxis leads to an increase in antibiotic resistance, as patients typically receive cycles of injections, and some of them receive a dozen or even several dozen injections. To date, there are studies proposing alternative substances, for example, preservative-free 0.6% povidone-iodine eye drops, as a perioperative prophylactic treatment for reducing the conjunctival bacterial load in patients undergoing intravitreal injection [15].

As demonstrated by Adebayo et al., there was no resistance to vancomycin in any Gram-positive bacteria isolated from the conjunctiva described in their study [16]. Currently, there are reports of endophthalmitis caused by Gram-positive bacteria with reduced susceptibility and/or vancomycin resistance [17].

Even if sterility conditions are maintained during intravitreal injections, there is no guarantee of avoidance of pathogen invasion. As reported by Garg et al., the prohibition of talking during medical procedures contributed to a reduction in the endophthalmitis incidence. They also reported a twofold lower number of all endophthalmitis cases in general and a sevenfold lower incidence of cases caused by oral pathogens (from 0.015% to 0.002%) [18].

While the pathogen causing the endophthalmitis in our series may have been introduced via contamination of the aflibercept injection, the drug lot and remaining syringes had a negative result for culture in all of the samples. A positive culture was obtained only from the patient’s bed and the surgeon’s seat.

Although *S. mitis/oralis* are still regarded as part of the physiological human oral flora, they can cause huge tissue damage in particular environments. Goldberg et al. described a series of twelve cases of endophthalmitis caused by this bacterium after intraocular bevacizumab injections. According to the report, patients with endophthalmitis manifestation were first treated with intravitreal antibiotic injections, and total vitrectomies were performed next. Overall, 10 of the 12 microbiological samples were positive for *S. mitis/oralis*.

In this case series, specimens from unused syringes with bevacizumab were cultured for bacteria and the outcomes were positive. Despite the prompt interventions, the four-month follow-up showed that three patients underwent evisceration or enucleation at the end of the therapy [19]. In our case series, no eye was enucleated in the nine-month follow-up, although the functional outcomes were very poor. *S. mitis/oralis* were described as devastating eye pathogens by Matthews et al. They reported 12 post-injection endophthalmitis cases caused by these bacteria, seven of which ended with the loss of the infected eye. The average time between the acute endophthalmitis phase and the enucleation was approximately five months (average 139.1 days) [6]. In our series of cases, the follow-up period was longer than nine months after the acute endophthalmitis, which led to optimistic conclusions regarding the therapeutic success.

The two aforementioned articles showed that the long-term follow-up turned out to be unsuccessful for patients who required eye removal. In our department, no patients presented symptoms of continuation or relapse of the ongoing inflammation. Because our follow-up period is substantially longer compared to the cited reports, this hopefully predicts an optimistic end result of the therapy.

Among all the patients with endophthalmitis after intravitreal injection, endophthalmitis caused by *Streptococcu*s species is associated with poorer visual acuity outcomes than endophthalmitis caused by coagulase-negative *Staphylococcu*s and culture-negative cases [13]. A study conducted by Yospaiboon over four years ago described visual acuity improvement in only 20% of patients with streptococcal endophthalmitis [20].

Only four of the seven patients in our case series were culture positive. In his review article, McCannel described that 48.0% of the samples from 50 cases of endophthalmitis were culture negative, and 52% were culture positive, from which one-third presented *Streptococci* [21]. The same situation was observed in our case series of post-injection endophthalmitis, where 42.9% of the collected material samples had a negative bacterial culture outcome. Therefore, empirical treatment was chosen as the therapy method, based on the knowledge of the etiology of the endophthalmitis.

As described in our report, vancomycin was used as the first-line drug in all cases. There is evidence in the literature proving the validity of vancomycin use in acute endophthalmitis. Marquart et al. reported that all *Streptococcus mitis/oralis* that were the cause of endophthalmitis were sensitive to vancomycin, whereas 77% of the strains were resistant to amikacin, and 27% of the strains were intermediately resistant to ceftazidime [22].

Vancomycin infusion during vitrectomy has been used both in postoperative and posttraumatic endophthalmitis, as described by Rejdak et al. [12]. In their study, the results of 45 patients were included. Visual acuities improved in 38 cases and were 1.0 logMAR in the group with postoperative endophthalmitis and 1.3 logMAR in the group with posttraumatic endophthalmitis; 44% of the cases were culture-positive (*Staphylococcus*, *Streptococcus*, *Enterococcus*, and *Bacillus* spp.).

Some authors suggested that the insertion of silicone oil might supplement the antimicrobial activity of intravenous antibiotics [23]. As reported by Relhan, silicone oil, systemic and topical antimicrobials, and intravitreal steroids were useful and important additions to the therapy [3]. The use of silicone oil facilitates better control of infection, anatomical stabilization, and a better final BCVA [24]. Ozdamar et al. reported that silicone oil had antimicrobial activity against *S. aureus*, *S. epidermidis*, *Pseudomonas aeruginosa*, *Candida albicans*, and *Aspergillus* spp., which are common endophthalmitis-causing agents [25].

The antimicrobial effectiveness of heavy silicone oil and conventional silicone oil against the compared endophthalmitis-causing microorganisms showed that conventional silicone oil decreased the colony number of all bacteria, except for *C. albicans*. Heavy silicone oil exerted a superior antimicrobial effect on all pathogens, including *C. albicans* [26]. In our series, all the patients required vitrectomy with silicone oil as a tamponade. In one patient, no silicone oil was given as primary treatment after vitrectomy, which led to a strong inflammatory reaction in the vitreous cavity within a short postoperative period (two days).

The patient underwent the second vitrectomy with a silicone oil endotamponade, resulting in no further inflammation recurrence or complications. This situation confirmed that, in the case of postoperative endophthalmitis, silicone oil should be applied immediately and obligatorily, as it blocks the strong inflammatory reaction caused by aggressive pathogens. Endophthalmitis is an urgent condition that requires prompt surgical treatment to achieve the best possible vision outcomes. Patients who underwent early vitrectomy (less than three days) showed the most favorable visual outcomes compared to those who underwent delayed surgery [20]. Kurniawan, however, reported that early vitrectomy within 48 h of presentation had no correlation with the visual outcome [27]. An additional effect of a silicone oil endotamponade is the prevention of postoperative retinal detachment [28].

The OCT results of eyes filled with silicone oil in our case series showed disruption of the inner retinal layers but stabilization of the retina in regard to changes related to wet age-related macular degeneration (AMD). This is consistent with the results obtained recently by Uhr et al. [29]. They concluded that endophthalmitis after anti-VEGF injection was associated with the relative stability of the underlying exudation. Lu et al. described the OCT results of 45 cases of eyes after endophthalmitis of different origins. The structural changes included inner segment ellipsoid disruption in 49% of the cases and atrophy of the retinal inner layers in 24%. The author concluded that changes of the retinal inner layers were likely caused by ischemia of the retina and were associated with visual impairment in endophthalmitis, despite successful management [30].

## 5. Conclusions

Our report shows that special care must be taken, and attention has to be paid to the preparation of the drugs and equipment prior to use in intraocular interventions.

A reduction in the incidence of infections involves compliance with procedures and hygiene; unfortunately, however, this complication can never be totally eliminated. Aggressive surgical intervention, prompted by clinical findings and vitreous humor cultures, is essential for saving both patients’ vision and their eyes.

## Figures and Tables

**Table 1:** Characteristics and treatment outcomes in patients with acute endophthalmitis after aflibercept injection (abbreviations: BCVA, best-corrected visual acuity; BSS, balanced salt solution; F, female; FC, finger counting; HM, hand movement; IOP, intraocular pressure; LP, light perception; M, male; NLP, no light perception; PPV, pars plana vitrectomy; * in patients after corneal epithelium abrasion).

No	Sex	Age	Microbiological Culture	Preoperative Treatment	Surgical Treatment	Postoperative Treatment	Initial BCVA	Final BCVA	Final Anterior Segment Status	Final Status of the Retina
1	M	83	Negative	Topical levofloxaxin drops	PPV + vancomycin + silicone oil + anterior chamber rinsing + corneal epithelium abrasion + cataract surgery	Topical: levofloxacin, dexamethasone, 1% atropine solution, dexpanthenol gel * Oral: levofloxacin 400 mg	LP	HM	Clear	Attached
2	M	82	*Streptococcus mitis/oralis*		1. PPV + vancomycin + anterior chamber rinsing + BSS tamponade 2. PPV + vancomycin + abrasion + anterior chamber rinsing + silicone oil tamponade		NLP	HM	PCO	Attached
3	F	60	Negative		PPV + vancomycin + silicone oil + anterior chamber rinsing + corneal epithelium abrasion + cataract surgery		LP	FC	Clear	Attached
4	F	56	Negative		PPV + vancomycin + anterior chamber rinsing + silicone oil tamponade + cataract surgery		HM	FC	Clear	Attached
5	M	62	*Streptococcus mitis/oralis*		PPV + vancomycin + anterior chamber rinsing + silicone oil tamponade + cataract surgery		HM	FC	Posterior synechiae	Attached
6	F	85	*Streptococcus mitis/oralis*		PPV + vancomycin + silicone oil + anterior chamber rinsing + corneal epithelium abrasion + cataract surgery		LP	HM	Keratic Precipitates	Attached
7	M	78	*Streptococcus mitis/oralis*		PPV + vancomycin + silicone oil + anterior chamber rinsing + corneal epithelium abrasion + cataract surgery		LP	FC	Clear	Attached

## Data Availability

The data presented in this study are available on request from the corresponding author.
